# In vivo development of resistance to novel β-lactam/β-lactamase inhibitor combinations in KPC-producing *Klebsiella pneumoniae* infections: a case series

**DOI:** 10.1007/s10096-024-04958-w

**Published:** 2024-10-10

**Authors:** Matteo Boattini, Gabriele Bianco, Sara Comini, Cristina Costa, Paolo Gaibani

**Affiliations:** 1 Department of Public Health and Paediatrics, University of Torino, Turin, Italy; 2 Microbiology and Virology Unit, University Hospital Città della Salute e della Scienza di Torino, Corso Bramante 88/90, Turin, 10126 Italy; 3 Lisbon Academic Medical Centre, Lisbon, Portugal; 4 Department of Experimental Medicine, University of Salento, Via Provinciale Monteroni n. 165, Lecce, 73100 Italy; 5 Operative Unit of Clinical Pathology, Carlo Urbani Hospital, Jesi, 60035 Italy; 6 Microbiology and Virology Unit, Azienda Ospedaliera Universitaria Integrata Di Verona, Verona, Italy; 7 Department of Diagnostics and Public Health, Microbiology Section, Verona University, Verona, Italy

**Keywords:** KPC, Ceftazidime/avibactam, Meropenem/vaborbactam, Imipenem/cilastatin/relebactam, Aztreonam/avibactam, Cefiderocol, *Klebsiella pneumoniae*

## Abstract

**Introduction:**

Understanding the dynamics that may characterize the emergence of KPC variants with resistance to novel β-lactam/β-lactamase inhibitor combinations (βL/βLICs) represents a challenge to be overcome in the appropriate use of recently introduced antibiotics.

**Methods:**

Retrospective case series describing development of multiple resistance to novel βL/βLICs in patients with KPC-producing *Klebsiella pneumoniae* (KPC-Kp) infections treated with these drugs. Clinical-microbiological investigation and characterization of longitudinal strains by Whole-Genome Sequencing were performed.

**Results:**

Four patients with KPC-Kp bloodstream infections were included. Most frequent clinical features were kidney disease, obesity, cardiac surgery as reason for admission, ICU stay, treatment with ceftazidime/avibactam, and pneumonia and/or acute kidney injury needing renal replacement therapy as KPC-Kp sepsis-associated complications. The development of resistance to ceftazidime/avibactam was observed in four longitudinal strains (three of which were co-resistant to aztreonam/avibactam and cefiderocol) following treatments with ceftazidime/avibactam (*n* = 3) or cefiderocol (*n* = 1). Resistance to meropenem/vaborbactam and imipenem/cilastatin/relebactam was observed in one case after exposure to ceftazidime/avibactam and imipenem/cilastatin/relebactam. Resistome analysis showed that resistance to novel βL/βLICs was related to specific mutations within *bla*_KPC_ carbapenemase gene (D179Y mutation [KPC-33]; deletion Δ242-GT-243 [KPC-14]) in three longitudinal strains, while porin loss (truncated OmpK35 and OmpK36 porins) was observed in one case.

**Conclusion:**

Therapy with novel βL/βLICs or cefiderocol may lead to the selection of resistant mutants in the presence of factors influencing the achievement of PK/PD targets. KPC variants are mainly associated with resistance to ceftazidime/avibactam, and some of them (e.g. KPC-14) may also be associated with reduced susceptibility to aztreonam/avibactam and/or cefiderocol. Loss of function of the OmpK35 and OmpK36 porins appears to play a role in the development of resistance to meropenem/vaborbactam and/or imipenem/relebactam, but other mechanisms may also be involved.

**Supplementary Information:**

The online version contains supplementary material available at 10.1007/s10096-024-04958-w.

## Introduction

*Klebsiella pneumoniae* carbapenemase-producing *Klebsiella pneumoniae* (KPC-Kp) has been a major public health concern in many countries for several years given the associated burden of healthcare-associated infections and high rate of mortality [1]. However, the diagnostic and clinical landscape has changed in the recent years [2]. The implementation of surveillance and infection control programs in healthcare institutions and the introduction of rapid diagnostic tests in bacteriology laboratories have made possible to control and monitor the diffusion of KPC-Kp more quickly, tracking and possibly limiting the spread of multidrug resistant pathogens [3–6]. Since 2015 with different timing and modalities depending on the country, the therapeutic armamentarium against KPC-Kp infections has been improved due to the introduction into clinical practice of new β-lactamase inhibitor-including combinations [7–15]. Avibactam and vaborbactam are broad spectrum β-lactamase inhibitors with the ability to inhibit class A β-lactamases such as KPC. Their potent activity against KPC-Kp in combination with ceftazidime and meropenem, respectively, has already been reported in several in vitro [7, 9] and clinical studies [11–16]. In addition, there are currently evidence on in vitro activity against KPC producers of avibactam in combination with aztreonam [10], the use of this compound being recommended against metallo-β-lactamase producers. Similarly, the combination of the new class A β-lactamases inhibitor relebactam with imipenem/cilastatin/relebactam also exhibited excellent in vitro activity against KPC producers [8, 9] but evidence on its clinical efficacy is very limited [16]. Also, although not part of β-lactam/β-lactamase inhibitor combinations (βL/βLICs), the siderophore cephalosporin cefiderocol has been also marketed having shown excellent in vitro activity against KPC-producing Enterobacterales [17, 18].

Factors determining resistance to novel βL/βLICs have been shown to be due to different mechanisms, including mutations within β-lactamase genes, porin loss, overexpression of ESBL genes and/or a combination of the above mechanisms [9, 19]. In this context, the widespread use of ceftazidime/avibactam which was the first βL/βLIC to become available, contributed through selective pressure to the exponential spread of ceftazidime/avibactam-resistant KPC variants [20–26] that until then had only been reported sporadically [27, 28]. In particular, the emergence of ceftazidime/avibactam resistance in KPC-Kp has been reported to be more frequent especially in patients with prolonged and suboptimal exposure to different β-lactam molecules [29] resulting from conditions such as critical illness, obesity, respiratory tract as the source of infection, renal disease, and infection severity [20–23, 30]. Since understanding the dynamics that may characterize the emergence of KPC-Kp strains with complex resistance phenotypes represents a challenge to be overcome in the appropriate use of recently introduced antibiotics, we described the clinical and genomic trajectories of resistance development to novel βL/βLICs in four clinical cases of infections due to KPC-Kp and treated with novel antimicrobial molecules.

## Methods

### Study design

This retrospective case series included patients who suffered from a KPC-Kp bloodstream infection treated with a novel βL/βLICs (i.e. ceftazidime/avibactam, meropenem/vaborbactam, imipenem/cilastatin/relebactam, and aztreonam/avibactam) and then presented a relapse of infection caused by a KPC-Kp strain displaying resistance to one or more of these drugs. All patients were admitted during the period January 1st 2021 to December 31st 2023 at the University Hospital Città della Salute e della Scienza di Torino, Turin, Italy. Patient electronic medical records were used for collecting the following data: patient baseline clinical characteristics, reason for admission, KPC-Kp antimicrobial susceptibility profiles, antibiotic management, sepsis-associated complications, timeframe for resistance onset to novel βL/βLICs and/or cefiderocol, and outcome. In order to determine the mechanisms related to the development of resistance to the novel βL/βLICs, genomic comparison on KPC-Kp longitudinal strains isolated from the same patient was also conducted.

### Microbiological diagnostics

MALDI-TOF coupled to mass spectrometry (Bruker Daltonics, Germany) was used to identify *K. pneumoniae* isolates recovered from patients’ blood cultures. Antimicrobial susceptibility testing results were obtained using broth microdilution. Susceptibility to cefiderocol was performed using iron-depleted cation-adjusted Mueller-Hinton broth microdilution (Bruker Daltonics GmbH Co. KG, Bremen, Germany), daily fresh prepared broth microdilution panel in concentration range 0.03–32 mg/L and following EUCAST guidelines [31]. Antimicrobial susceptibility testing results were interpreted according current EUCAST clinical breakpoints (v. 14.0) [32]. Xpert Carba-R (Cepheid, Sunnyvale, CA, USA) was used to detect KPC gene in *K. pneumoniae* isolates.

### Whole genomic sequencing and genetic analysis

Whole-Genome sequencing and genomic data analysis were performed on longitudinal KPC-Kp strains collected from each patient before and during/after therapy as previously described [33]. Briefly, genomic DNA was sequences using Illumina Iseq100 platform (Illumina, San Diego, CA, USA) with a 2 × 150 paired end run and assemblies were performed with SPAdes v.3.10 with careful settings. Multi-locus sequence type was performed using PubMLST typing schemes v2.23.0 (https://github.com/tseemann/mlst). Resistome analysis and plasmid incompatibility type were performed using CGE server (https://cge.cbs.dtu.dk/services/; accessed on 1 Mar 2024), PlasmidFinder (https://cge.cbs.dtu.dk/services/PlasmidFinder/; accessed on 1 Mar 2024), respectively. Then, β-lactamases genes were manually investigated by BLAST analysis using the β-Lactamase Database (http://www.bldb.eu; accessed on 1 Mar 2024).

Single nucleotide variants, insertions and/or deletions in the genomes of longitudinal KPC-Kp strains were performed using BreSeq software, as previously described [34]. Clonal relatedness of the KPC-Kp strains was performed based on core genome SNPs analysis of the genomes derived from longitudinal strains collected from each patient and using Italian KPC-Kp genomes available in GenBank. Genome assemblies have been deposited in the NCBI database and are freely accessible.

## Results

**Fig. 1:**
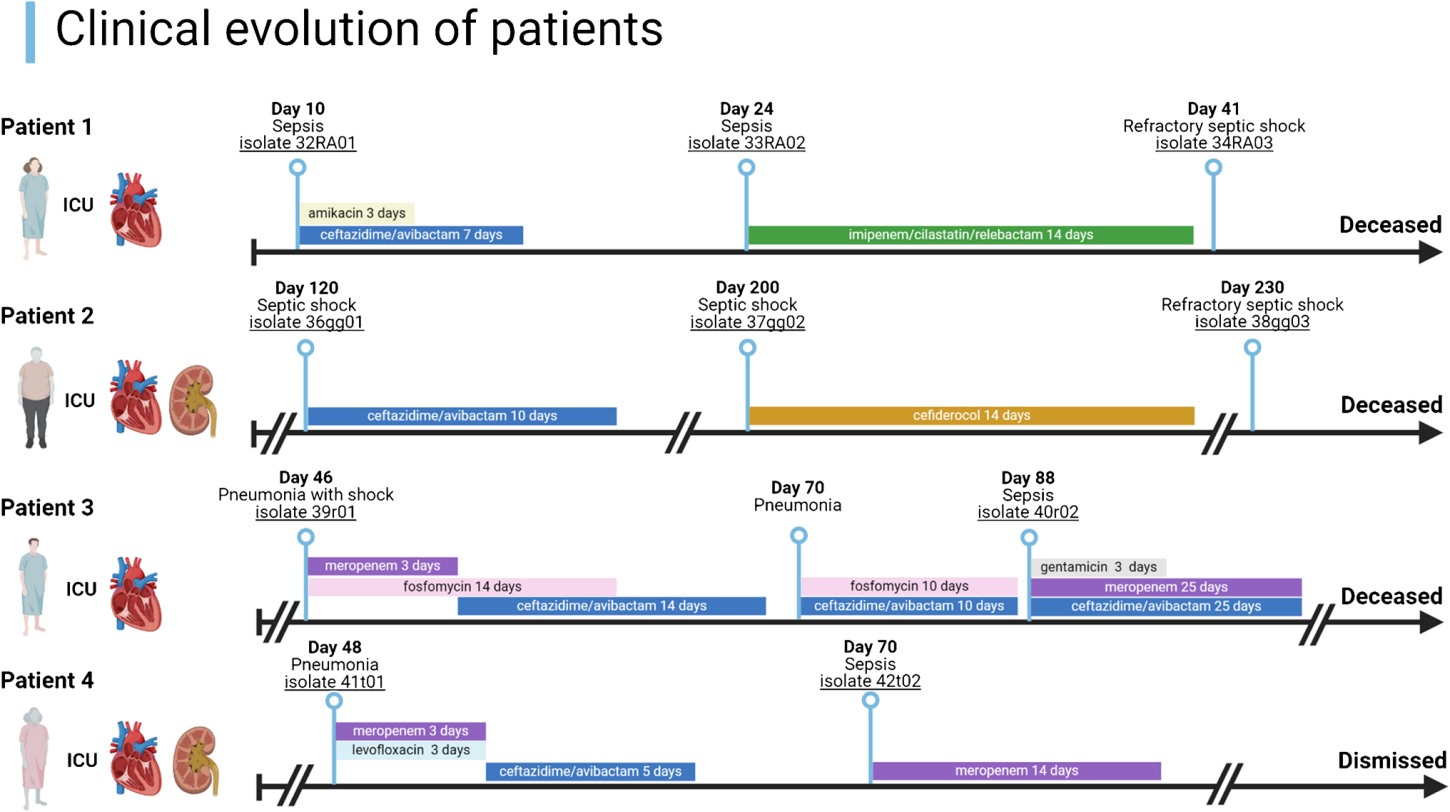
Clinical evolution of the patients included in the study (Created in BioRender. Boattini, M. (2024) BioRender.com/n78m272)

Four patients with a total of ten KPC-Kp bloodstream infections were included (Table 1). The clinical evolution of the patients included in the study was presented in Fig. 1. Antimicrobial susceptibility profiles of KPC-Kp strains included in the study were reported in Table 2. The cases of the included patients together with the genomic characterization of the longitudinal KPC-Kp clinical strains were presented below.

**Table 1:** Clinical features of patients suffering from KPC-producing *Klebsiella pneumoniae* bloodstream infection included in the study

Patient	Gender, Age (years)	Comorbidities	Reason for admission	KPC-Kp BSI isolate	Targeted antibiotic therapy	Sepsis-associated complications	Timeframe for CZA resistance (days)	Timeframe for MEV or IMR resistance (days)	Timeframe for ATZ/AVI resistance (days)	Timeframe for CFDC resistance (days)	Length of stay (days)	In-hospital death
1	F, 75	Atrial fibrillation Aortic bioprosthesis	Heart failure	32RA01	CZA+AK	AKI	14	-	-	-	42	Yes
				33RA02	IMR		-	14	-	-		
				34RA03	-		-	-	-	-		
2	M, 63	Obesity IHD ESRD	Solid organ transplantation	36gg01	CZA	AKI	-	-	-	-	233	Yes
				37gg02	CFDC		30	-	30	30		
				38gg03	-		-	-	-	-		
3	M, 62	Heart valve disease	Cardiac surgery	39r01	CZA+FF	HAP, AKI	42	-	42	-	138	Yes
				40r02	CZA+MEM+GM		-	-	-	-		
4	F, 69	Diabetes Heart failure CKD	LVAD implantation	41t01	CZA	VAP, AKI	22	-	22	-	148	No
				42t02	MEM		-	-	-	-		

*KPC-Kp* KPC-producing *Klebsiella pneumoniae*, *BSI* bloodstream infection, *CZA* ceftazidime/avibactam, *MEV* meropenem/avibactam, *IMR* imipenem/cilastatin/relebactam, *AZT/AVI* aztreonam/avibactam, *CFDC* cefiderocol, *AK* amikacin, *AKI* acute kidney injury, *IHD* ischemic heart disease, *ESRD* end-stage renal disease, *FF* fosfomycin, *MEM* meropenem, *GM* gentamicin, *HAP* hospital-acquired pneumonia, *CKD* chronic kidney disease, *LVAD* lef ventricular assist device, *VAP* ventilator-associated pneumonia

**Table 2:**
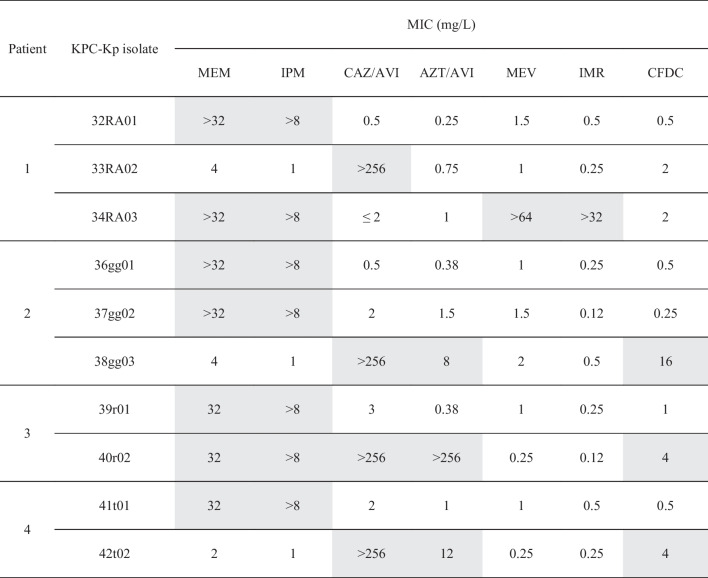
Antimicrobial susceptibility profiles of KPC-producing Klebsiella pneumoniae strains included in the study

Grey shading indicated drug-resistant strain according to EUCAST clinical breakpoints (v. 14.0)

*KPC-Kp* KPC-producing Klebsiella pneumoniae, *MEM* meropenem, *IPM *imipenem, *CAZ/AVI* ceftazidime/avibactam, *AZT/AVI* aztreonam/avibactam, *MEV* meropenem/avibactam, *IMR* imipenem/relebactam, *CFDC* cefiderocol

### Case 1

A 75-year-old woman with an aortic bioprosthesis for severe aortic regurgitation was admitted due to heart failure for bioprosthetic valve dysfunction. Valve reintervention was complicated by cardiogenic shock and acute kidney injury. Surveillance rectal swabs revealed that the patient was colonized by KPC-Kp. On day 10, patient presented with sepsis and blood cultures yielded a ceftazidime/avibactam-susceptible KPC-Kp strain (isolate 32RA01). Targeted antibiotic therapy with ceftazidime/avibactam (seven days) and amikacin (three days) was carried out with patient clinical improvement. On day 24, patient presented with a new episode of sepsis. Blood cultures yielded a ceftazidime/avibactam-resistant KPC-Kp strain (isolate 33RA02) and patient was treated with imipenem/cilastatin/relebactam during 14 days. On day 41, patient presented with an episode of refractory septic shock and died within a few hours. Blood cultures yielded a ceftazidime/avibactam-susceptible, meropenem/vaborbactam- and imipenem/cilastatin/relebactam-resistant KPC-Kp strain (isolate 34RA03).

### Case 2

A 63-year-old man was admitted for solid organ transplantation. His past medical history was remarkable for obesity, hypertensive/ischemic heart disease and end-stage chronic kidney disease. During the post-op period, patient was diagnosed with KPC-Kp rectal carriage, presented with prolonged weaning and several bacterial infections. During the last four months of hospitalization, patient presented with three KPC-Kp bloodstream infection episodes, the last of which was fatal. The first on day 120, characterized by septic shock, acute kidney injury requiring renal replacement therapy and sustained by a ceftazidime/avibactam-, aztreonam/avibactam-, and cefiderocol-susceptible KPC-Kp strain (isolate 36gg01), was treated with ceftazidime/avibactam during ten days with patient clinical improvement. The second about 80 days later, caused by a ceftazidime/avibactam-, aztreonam/avibactam-, and cefiderocol-susceptible KPC-Kp (isolate 37gg02), was treated with cefiderocol during 14 days with patient clinical improvement. The third 30 days later was characterized by a refractory septic shock sustained by a ceftazidime/avibactam-, cefiderocol- and aztreonam/avibactam-resistant KPC-Kp strain (isolate 38gg03).

### Case 3

A 62-year-old man with hypertensive cardiomyopathy and severe mitral valve regurgitation was admitted for mitral valve replacement. Cardiac surgery was complicated by cardiogenic shock due to anterolateral acute myocardial infarction, prolonged weaning, and several bacterial infections. Surveillance rectal swabs during ICU admission revealed that the patient was colonized by KPC-Kp. Among the infections that occurred during hospitalisation, to be highlighted that on day 46 patient presented with sepsis due to hospital-acquired pneumonia that contributed to the onset of oliguric acute kidney injury requiring renal replacement therapy. Empirical therapy with meropenem, intravenous fosfomycin and daptomycin was started. Bronchoalveolar lavage fluid and blood cultures yielded a ceftazidime/avibactam- and aztreonam/avibactam-susceptible KPC-Kp strain (isolate 39r01), and the antibiotic therapy was changed to ceftazidime/avibactam plus intravenous fosfomycin for 14 days with patient clinical improvement. On day 70, patient presented with an another episode of hospital-acquired pneumonia with detection of a ceftazidime/avibactam- and aztreonam/avibactam-susceptible KPC-Kp strain on bronchoalveolar lavage fluid that was successfully treated with ceftazidime/avibactam and intravenous fosfomycin during 10 days. On day 88, patient presented with sepsis and blood culture yielded a ceftazidime/avibactam- and aztreonam/avibactam-resistant KPC-Kp (isolate 40r02). Meropenem, ceftazidime/avibactam and gentamicin were started for an overall therapy of 25 days with patient clinical improvement. Several infectious complications followed, leading to multi-organ failure and death 40 days later.

### Case 4

A 69-year-old woman with diabetes, chronic kidney disease and NYHA class IV ischemic heart failure was admitted for a left ventricular assist device implantation. Surveillance rectal swabs after implantation during ICU admission revealed that patient was colonized by KPC-Kp. The post-implantation period was complicated by prolonged weaning and ventilator-associated pneumonia with septic shock and oliguric acute kidney injury requiring renal replacement therapy. Empirical therapy with meropenem, levofloxacin and daptomycin was started. Blood cultures yielded a ceftazidime/avibactam- and aztreonam/avibactam-susceptible KPC-Kp strain (isolate 41t01), and the antibiotic therapy was changed to ceftazidime/avibactam (five days). Approximately three weeks later, the patient presented a new episode of sepsis and blood cultures yielded a ceftazidime/avibactam- and aztreonam/avibactam-resistant KPC-Kp strain (isolate 42t02). Antibiotic therapy with meropenem was started according to antimicrobial susceptibility testing results. The patient experienced several other complications but managed to be discharged to a rehabilitation facility after almost six months of hospitalisation.

### Genomic characterization of KPC-Kp strains

Genetic characteristics of KPC-Kp isolates included in this study were summarized in Table 3, while whole resistome and genomic analysis were shown in Table S1 (supplementary material). MLST analysis showed that in three of the four clinical cases, strains collected from the same patient belonged to an identical sequence type (ST). In case no. 3, 39r01 and 40r02 isolates exhibited a single SNP to the *rpoB* locus belonging to closely related STs (i.e. ST512 and ST868) within the clonal complex CC512. At the same time, phylogenetic analysis showed that isolates from the same patient cluster closely together in the phylogenetic tree, being genomically closely related (Fig. S1, supplementary material). Analysis of plasmid replicons revealed that isolates collected from the same patient before and during/after treatment had the same plasmid profile in all KPC-Kp. The resistome analysis of the longitudinal KPC-Kp strains showed that, in three of the four clinical cases (i.e. cases no. 1, 2 and 4), resistance to ceftazidime/avibactam was related to specific mutations within *bla*_KPC_ carbapenemase genes. In detail, genome comparison showed no significant differences in β-lactamase genes with the exception of the carbapenemase genes which exhibited a single SNPs of *bla*_KPC−2_ (case no. 1, D179Y mutation [KPC-33]; case no. 2, deletion Δ242-GT-243 [KPC-14]; case no. 4, [KPC-14]). In case no. 3, however, no differences were observed within *bla*_KPC−3_ carbapenemase gene, whereas genetic variation was detected in the porin genes. In particular, sequence analysis of porin-encoding genes revealed that 40r02 isolate collected after ceftazidime/avibactam-based treatment harbored a truncated OmpK35 and OmpK36 porins, while parental strain (i.e. 39r01) collected before antimicrobial therapies carried a truncated OmpK35 and wild-type OmpK36 with an insertion of GD at position 135 (Table 3). Similar results were observed when comparing the readings of the KPC-Kp strains with those of the parental strains collected from the same patient, showing that most of the SNPs were located within hypothetical or phage-related proteins (Table S2, supplementary material).

**Table 3:** Genomic characteristics of the KPC-producing *Klebsiella pneumoniae* longitudinal isolates collected from the four different patients

Patient	KPC-Kp isolate	Resistance phenotype	MLST	β-lactamases	Efflux pumps	Porins	Plasmid replicons
1	32RA01	MEM IPM	101	KPC-2 SHV-1	EmrD OqxA OqxB	OmpK35 wt OmpK36 ins135GD	ColRNAI IncFIA(HI1)
	33RA02	CAZ/AVI	101	KPC-33 SHV-1	EmrD OqxA OqxB	OmpK35 wt OmpK36 ins135GD	ColRNAI IncFIA(HI1)
	34RA03	MEM IPM MEV IMR	101	KPC-2 SHV-1 TEM-1	EmrD OqxA OqxB	OmpK35 wt OmpK36 ins135GD	ColRNAI Col156 Col(MG828) IncFIA(HI1) IncFII(K)
2	36gg01	MEM IPM	101	KPC-2 SHV-1	EmrD OqxA OqxB20	OmpK35 wt OmpK36 ins135GD	ColRNAI IncFIA(HI1)
	37gg02	MEM IPM	101	KPC-2 TEM-1 SHV-1	EmrD OqxA OqxB20	OmpK35 wt OmpK36 ins135GD	ColRNAI IncFIA(HI1)
	38gg03	CAZ/AVI AZT/AVI CFDC	101	KPC-14 SHV-1	EmrD OqxA OqxB20	OmpK35 wt OmpK36 ins135GD	ColRNAI Col(MG828) IncFIA(HI1) IncFII(K)
3	39r01	MEM IPM	512	KPC-3 TEM-1 SHV-11	EmrD OqxA OqxB	OmpK35 truncated at aa 88 OmpK36 ins135GD	ColRNAI IncFIB(K) IncFIB(pQIL) IncFII(K)
	40r02	MEM IPM CAZ/AVI AZT/AVI CFDC	868	KPC-3 SHV-11	EmrD OqxA OqxB	OmpK35 truncated at aa 88 OmpK36 truncated at aa 81	ColRNAI IncFIB(K) IncFIB(pQIL) IncFII(K)
4	41t01	MEM IPM	1685	KPC-2 TEM-206 SHV-1 OXA-1 CTX-M-15	EmrD OqxA OqxB20	OmpK35 wt OmpK36 ins135GD	ColRNAI IncFIA(HI1) IncFIB(K) IncFII(K) IncR
	42t02	CAZ/AVI AZT/AVI CFDC	1685	KPC-14 TEM-206 SHV-1 OXA-1 CTX-M-15	EmrD OqxA OqxB20	OmpK35 wt OmpK36 ins135GD	ColRNAI IncFIA(HI1) IncFIB(K) IncFII(K) IncR

*KPC-Kp* KPC-producing *Klebsiella pneumoniae*, *MLST* MultiLocus Sequence Type, *wt* wild type, *MEM* meropenem, *IPM* imipenem, *CAZ/AVI* ceftazidime/avibactam, *AZT/AVI* aztreonam/avibactam, *MEV* meropenem/avibactam, *IMR* imipenem/relebactam, *CFDC* cefiderocol

Lastly, genomic analysis showed that all KPC-Kp strains harbored Tn4401 “isoform a” and the absence of specific SNPs within the promoter region of the *bla*_KPC_ gene (Table S1, supplementary material).

## Discussion

This study offered a clinical and genomic insight into the i*n vivo* development of resistance to novel βL/βLICs in patients with KPC-Kp bloodstream infection treated with these drugs. What emerged revealed the relevance in this context of clinical features such as kidney disease, obesity, cardiac surgery as reason for admission, ICU stay, previous treatment with ceftazidime/avibactam, and pneumonia or acute kidney injury needing renal replacement therapy as KPC-Kp sepsis-associated complications. In particular, the development of resistance to ceftazidime/avibactam was observed in four longitudinal strains (three of which were co-resistant to aztreonam/avibactam and cefiderocol) following treatment with ceftazidime/avibactam or cefiderocol. At the same time, cross-resistance to meropenem/vaborbactam and imipenem/cilastatin/relebactam was observed in one case following exposure to ceftazidime/avibactam and imipenem/cilastatin/relebactam. The resistome analysis showed that resistance to novel βL/βLICs was related to specific mutations within *bla*_KPC_ carbapenemase gene (KPC-33 and KPC-14) or to wild type bla_KPC−3_ gene in association with genetic variation in the porin genes (truncated OmpK35 and OmpK36 porins).

The profile of patients who developed resistance to one or more of novel βL/βLICs included in this study is consistent with currently available evidence that identifies critical illness, renal disease, and the type of renal replacement therapy [35] as some of the conditions that may increase the risk for underdosing antimicrobial agents and selecting resistant strains. Obesity, which affected only one of the patients of this study, is frequently reported among the factors affecting PK/PD targets attainments of β-lactams but its impact in this process appears to be less substantial [36]. A similar consideration must also be made for patients admitted for cardiac surgery and possibly undergoing ECMO, the evidence on whose impact is still limited [36]. In contrast, the impact of the use of ceftazidime/avibactam in the selection process of novel βL/βLICs resistant strains, is certainly more marked, especially in the case of respiratory infection KPC-Kp sepsis-associated complication. We can speculate that the fact that previous suboptimal exposure to ceftazidime/avibactam is one of the most frequent factors in the selection process of KPC-Kp strains resistant to the novel βL/βLICs might be mainly due to two reasons. First, ceftazidime/avibactam is the drug first introduced into clinical practice and most widely used. However, our study showed two cases of resistance to novel βL/βLICs following imipenem/cilastatin/relebactam and cefiderocol treatment, depriving ceftazidime/avibactam of the exclusivity of inducing resistance. Second, the suboptimal ability of ceftazidime/avibactam to penetrate the lungs was associated with high clinical and microbiological failure [37, 38], and exposure to this antibiotic seems to induce resistant strains more easily in the respiratory tract and in the blood than in the intestinal tract [30].

Our study also showed that the main mechanism of resistance to ceftazidime/avibactam was the expression of KPC variants, as opposed to the wild-type gene that is usually susceptible [27]. Indeed, amino acid substitutions and/or insertions and/or deletions modifying functional properties in the Ω-loop hydrogen bonding structure or other protein regions of the KPC enzyme represent the most widespread mechanism of ceftazidime/avibactam resistance in KPC-Kp especially in derivative of *bla*_KPC−2_ or *bla*_KPC−3_ [27]. These alterations may lead to poor inhibition by avibactam, increased ceftazidime hydrolysis, reduced catalytic activity of carbapenems – especially imipenem [39], and reduced susceptibility to cefiderocol [40–42]. Our findings showed that most of these characteristics were found in isolates 38gg03 and 42t02 characterized by KPC-14 of clinical case no. 2 and 4, respectively. Cloning experiments in *Escherichia coli* have indeed shown that KPC-14 is characterized by increased affinity for ceftazidime, loss of activity towards carbapenems, and no impact on affinity for aztreonam [43]. Steady-state kinetics and molecular modelling studies confirmed that KPC-14 has a higher affinity for ceftazidime and reduced activity against carbapenems (40-fold and 200-fold lower than KPC-2, respectively) [43]. These characteristics therefore might provide an explanation for cross-resistance to ceftazidime/avibactam and cefiderocol (due to structural similarity with ceftazidime), resistance to aztreonam/avibactam, and restoration of susceptibility to carbapenems.

Other resistance mechanisms to novel βL/βLICs include increased *bla*_KPC_ copy number on plasmids leading to bacterial tolerance to β-lactams and greater possibility of mutation occurrence as well as outer membrane porin deficiencies with disruption in *ompK35*, *ompK36*, and *ompK37*. These mechanisms have been reported to be associated with development of cross-resistance to ceftazidime/avibactam, meropenem/vaborbactam, and imipenem/cilastatin/relebactam [33, 44–48]. Although we did not analyze, increased *bla*_KPC_ copy number might be involved as resistance mechanism in 34RA03 isolate. In addition, our study detected genetic variation in the porin genes in the 40r02 strain resistant to ceftazidime/avibactam, aztreonam/avibactam, and cefiderocol.

The strength of this study is to outline clinically and genomically the development of multiple resistance to novel βL/βLICs to investigate a non-negligible knowledge gap that should certainly be the subject of future research. Some limitations should be also acknowledged, including its retrospective nature and the fact that it was conducted in a single center. The narrow nature of our findings, due to the limited sample size, does not allow generalized conclusions to be drawn. At the same time, it was not possible to exclude that large genomic rearrangement occurred in longitudinal strains by using short-reads approach. In this context, further experiments will be performed using long reads to completely characterize the chromosome and plasmids architectures of strains collected from the same patient.

In conclusion, our study revealed that resistance developed to ceftazidime/avibactam was predominantly due to KPC variants selected during therapy in patients with clinical conditions that may alter exposure to the drug. In addition, KPC-14 was associated with resistance towards both ceftazidime/avibactam, aztreonam/avibactam and cefiderocol. Conversely, multiple mechanisms (i.e. porins truncation) may be related to the emergence of resistance to meropenem/vaborbactam and imipenem/cilastatin/relebactam. The judicious use of βL/βLICs based on the individual patient risk stratification of developing resistance to these new drugs, together with surveillance programs, currently appear to be the most relevant actions to be reinforced to reduce the impact of the circulation of KPC-Kp strains with complex resistance phenotypes.

## Supplementary information

Below is the link to the electronic supplementary material.

ESM 1 (DOCX 63.9 KB)

ESM 2 (XLSX 39.0 KB)

ESM 3 (XLSX 157 KB)

## Data Availability

The authors confirm that the data supporting the findings of this study are available upon reasonable request.
