# Supplementary material for: In vivo development of resistance to novel β-lactam/β-lactamase inhibitor combinations in KPC-producing Klebsiella pneumoniae infections: a case series
Source: Eur J Clin Microbiol Infect Dis. 2024 Oct 10;43(12):2407–17. doi: 10.1007/s10096-024-04958-w (PMC11608324; doi:10.1007/s10096-024-04958-w)

**Figure S1**. Phylogenetic tree of longitudinal KPC-producing *Klebsiella pneumoniae* strains included in this study (colored in red). Phylogenetic analysis was performed based on core genome SNPs.


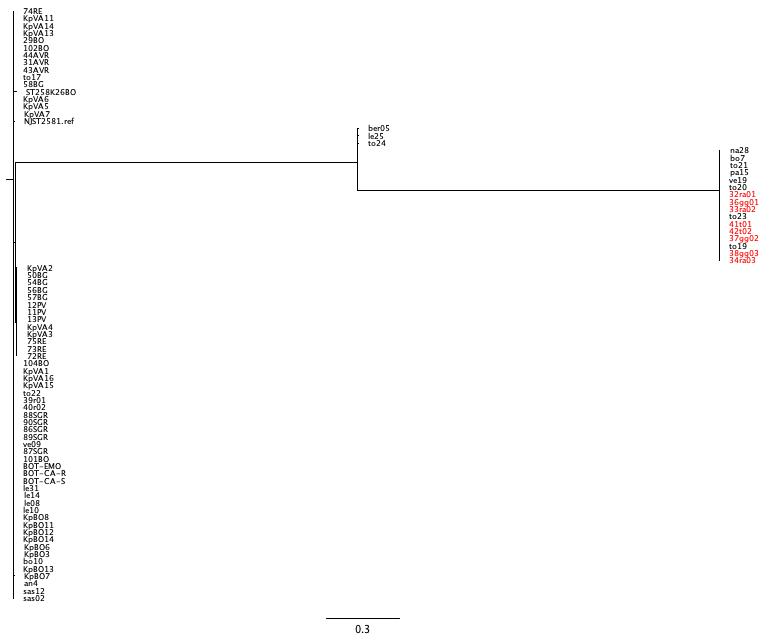

Supplement: Supplementary file 1 — (DOCX 63.9 KB) [file 10096_2024_4958_MOESM1_ESM.docx]
